# Meningiomas in Elderly Patients: Epidemiology, Clinical Characteristics, Prognostic Factors, and Personalized Treatment Strategies

**DOI:** 10.31083/RN53896

**Published:** 2026-09-22

**Authors:** Shuaishuai Wu, Jingjing Wang, Liu Zhang, Xiangji Meng, Yanfen Yao, Haiyan Jia, Zongxu Sun, Yahu Bai, Haixia Jin, Lingzhi Li, Changli Wang

**Affiliations:** ^1^Department of Intensive Care Medicine, Shandong Provincial Third Hospital, Shandong University, 250100 Jinan, Shandong, China; ^2^Department of Pathology, Shandong Cancer Hospital and Institute, Shandong First Medical University and Shandong Academy of Medical Sciences, 250117 Jinan, Shandong, China; ^3^Department of Neurosurgery, The First Affiliated Hospital of Jinan University, 510632 Guangzhou, Guangdong, China; ^4^Department of Neurosurgery, Shandong Cancer Hospital and Institute, Shandong First Medical University and Shandong Academy of Medical Sciences, 250117 Jinan, Shandong, China

**Keywords:** meningioma, aged, prognosis, frailty, treatment outcome

## Abstract

As the global population ages, the incidence of meningiomas rises, and the demand for clinical management of elderly patients also rises. Meningiomas are common intracranial tumors, and their incidence increases with age. Elderly patients with meningiomas are prone to comorbidities, reduced reserve function, atypical manifestations, increased perioperative risks, and impaired functional recovery, making management more difficult. The application of neuroimaging leads to more elderly patients with asymptomatic or incidentally discovered meningiomas. Choosing the best strategy among monitoring, surgery, radiotherapy, and other strategies is therefore key to clinical decision-making. For asymptomatic, small, and radiologically low-risk meningiomas in elderly patients, active surveillance is generally the preferred initial strategy. Surgery is more appropriate for symptomatic tumors, lesions with mass effect, documented growth, progressive peritumoral edema, neurological deterioration, or other high-risk clinical and radiological features. However, the treatment goal for elderly patients should emphasize symptom relief, preservation of neurological function, and quality of life more than pursuing gross total resection as routine. Stereotactic radiosurgery and fractionated radiotherapy provide important supplementary treatments for very elderly patients, high surgical risk patients, and postoperative residual lesions. This article reviews recent advances in understanding meningiomas in elderly and very elderly patients, focuses on epidemiology, clinical characteristics, prognostic factors, and treatment strategies, and also discusses the core principles of individualized management for this population to guide clinical practice.

## 1. Introduction

In recent decades, improvements in public health, chronic disease management, and social welfare systems have prolonged global life expectancy and contributed to an aging population [1]. According to the United Nations, the population aged 65 years and older is projected to increase substantially by 2050, with the population aged 80 years and older reaching 426 million [2,3]. Consequently, the incidence and medical burden of age-related neurological diseases are rising, with meningioma being one of the most relevant conditions.

Meningiomas arise from arachnoid cap cells and are among the most common primary intracranial tumors, accounting for approximately one-third of all central nervous system tumors [4,5]. Although most meningiomas are World Health Organization (WHO) grade I lesions, they exhibit substantial heterogeneity in growth rate, biological behavior, recurrence risk, and clinical presentation [6,7]. Meningiomas can occur at any age, but their incidence increases significantly with age, especially in middle-aged and elderly people [8,9]. With the widespread use of magnetic resonance imaging (MRI) and other neuroimaging modalities, an increasing number of elderly patients are now diagnosed with incidental meningiomas in the absence of overt neurological symptoms [10].

However, the management of elderly patients with meningiomas cannot be directly applied to young adults. Elderly patients have conditions such as hypertension, coronary heart disease, diabetes, cerebrovascular disease and chronic lung disease. They may also be accompanied by weakness, malnutrition, cognitive impairment, functional dependence and other conditions [11,12,13]. These factors will affect perioperative risks, treatment tolerance, and long-term functional recovery and require careful balancing among tumor control, complication avoidance, and quality-of-life assurance. Especially for asymptomatic lesions, skull base tumors, and elderly patients, there is no universally accepted standard for selecting between observation, surgery, and radiotherapy [6,14]. Therefore, a comprehensive understanding of the epidemiology, clinical manifestations, prognostic factors, and treatment strategies for meningiomas in elderly patients is essential for improving individualized management in this growing population.

## 2. Epidemiological Characteristics

Meningioma accounts for a substantial proportion of adult intracranial tumors and is the most common nonglial primary brain tumor [14,15]. Epidemiological studies have consistently shown that both incidence and prevalence increase with age, peaking in individuals aged 55–89 years [7,16,17]. In the general population, the incidence of meningioma is approximately 1 in 12,500, whereas in individuals older than 80 years, it may increase to around 1 in 2000 [17]. This pattern underscores advanced age as a major epidemiological context for meningioma development.

A study by Kuratsu et al. [18] on intracranial tumors found that meningiomas accounted for 32.2% (504 cases), of which 38.9% (196 cases) were incidental findings; patients aged 70 and older accounted for 31.7% of all meningioma cases, of which 49.3% (79 cases) were incidental findings. Bateman et al. [19] used the US Inpatient Database, including 8861 patients who underwent meningioma resection, and reported that approximately 26% were over 70 years old. Vernooij et al. [20] conducted a population-based MRI survey of 2000 volunteers and found that the prevalence of meningiomas was 0.5% among those aged 45–59, 1% among those aged 60–74, and 1.6% among those aged 75–97. With the increasing use of neuroimaging and the aging population, the proportion of elderly meningiomas in neurosurgery’s workload has become increasingly significant.

Recent studies indicate that this trend is intensifying. Amoo et al. [21] conducted a review noting that the incidence of meningiomas in the 65–69 age group is approximately 25–30 cases per 100,000 people, and that with increasing age, the incidence among those aged 85 and older may reach 50 cases per 100,000. Approximately 80%–90% of meningiomas are benign (WHO I), 15%–20% are atypical (WHO II), and less than 5% are malignant/anaplastic (WHO III). The incidence of atypical or malignant meningiomas appears to be higher in the elderly, and meningiomas in the elderly should not all be considered low-risk benign tumors [22,23,24].

There are significant gender differences in the incidence of meningioma, which may be related to hormonal mechanisms. Previous studies have found that the overall incidence of meningioma in women is approximately twice that in men, and this ratio appears to increase with age. Withrow et al. [25] analyzed the overall trend level of nonmalignant meningiomas using Surveillance, Epidemiology, and End Results (SEER) data from 2004 to 2017, and found that radiologically confirmed diagnoses were more common in elderly patients and patients with smaller tumors, and the incidence rate increased with age. But this pattern is not so obvious in malignant meningiomas. In addition, many asymptomatic small meningiomas in the elderly are treated non-surgically, and pathologically based epidemiological estimates may underestimate the true disease burden. With imaging and clinical diagnosis being more integrated into routine practice, the actual detection rate of meningiomas in older people may continue to rise.

## 3. Clinical Characteristics

Elderly patients with meningiomas have an insidious onset and a relatively long course, and are more likely to have atypical manifestations than young adults [26]. Older adults often have brain atrophy, and the intracranial compensatory space is relatively large. Even slowly growing large tumors may have no clinical symptoms for a long time, and obvious increased intracranial pressure or focal neurological deficits will appear only in the late stage. Therefore, some elderly patients are diagnosed only when the tumor is large, and present with mild headache, memory loss, behavioral changes or cognitive impairment [26,27]. These manifestations are easily mistaken for cerebrovascular diseases, neurodegenerative diseases, depression or dementia.

Symptoms of increased intracranial pressure in elderly patients often appear relatively late. When nausea, vomiting, or papilledema occurs, the tumor often already has an occupying effect [27]. Focal neurological deficits correlate with the tumor’s location. Lesions in important brain function areas may cause hemiplegia, sensory disturbances, aphasia, or visual field defects; due to differences in compensation and slow tumor growth, these symptoms in older people may be hidden in the early stage [26]. Frontal lobe or parasagittal sinus meningiomas often present initially with behavioral/personality changes, and it is necessary to recognize cognitive/psychiatric symptoms in this population.

Cognitive dysfunction in patients with meningiomas has gradually become a major clinical problem [6,28]. Studies have shown that these patients may have impairments in executive function, processing speed, and language fluency [29]. This situation is more complex in older adults, because aging itself is a major risk factor for cognitive decline. Epidemiological studies indicate that the background prevalence of mild cognitive impairment is 6.7% among patients aged 60–64, and rises to 25.2% among patients aged 80–84 [30]. The multiple factors of cognitive impairment in elderly patients with meningioma may not only reflect the tumor location and volume, but also be accompanied by neurodegeneration or cerebrovascular disease. It should be noted that small incidental meningiomas generally do not cause measurable cognitive impairment [31].

Seizures are relatively common, about 30% of patients have this condition, especially among patients with convexity meningiomas [32]. Seizures in older people may be atypical, such as transient confusion, falls, abnormal behavior, or non-convulsive or subtle seizure manifestations; these conditions may be mistaken for transient ischemic attack (TIA), delirium or cognitive impairment [6]. Tumors in the dominant cerebral hemisphere can cause language disorders; meningiomas at the cerebellopontine angle may cause hearing loss, tinnitus, or an unsteady gait, but prior degenerative changes or pre-existing neurological diseases often mask these symptoms in the elderly [33]. Therefore, for elderly patients with meningiomas, clinical evaluation should combine detailed neurological examination with high-quality imaging.

## 4. Prognostic and Recurrence-Related Factors

The prognosis for elderly patients with meningiomas is influenced by several interconnected factors, including the tumor’s pathological or radiological characteristics, the patient’s overall health status, and their ability to tolerate treatment [7,10,34,35,36,37]. Previous studies have shown that several factors are associated with poorer outcomes, including advanced age, male sex, larger tumor size, significant peritumoral edema, lower preoperative Karnofsky Performance Status (KPS), skull base tumor location, WHO II–III histology, incomplete surgical resection, and perioperative complications [7,10,34,35,36,37].

Although postoperative survival rates are generally lower in elderly meningioma patients in certain cohorts, chronological age alone should not be considered a poor prognostic factor [7,34,35]. Current evidence suggests that the poorer outcomes observed in elderly patients are more likely to reflect age-related biological changes, reduced physiological and organ reserve, increased frailty, and a higher burden of comorbidities—all of which may affect postoperative recovery and treatment tolerance [12]. Notably, previous studies have shown that among appropriately selected elderly patients, long-term survival rates following meningioma surgery may be comparable to those of an age- and sex-matched general population, and that functional status, comorbidity, tumor biology, and extent of resection are more relevant prognostic determinants than chronological age alone. Therefore, prognostic assessment for meningiomas should go beyond age as an isolated variable and instead incorporate a more comprehensive evaluation of functional capacity, general health, frailty, and tumor-related characteristics.

Li et al. [38] conducted a comprehensive analysis of the prognosis of elderly patients who underwent meningioma resection. The study included 478 elderly participants, of whom 47 (10.0%) died during follow-up. Analysis revealed that the 3-, 5-, and 10-year overall survival (OS) rates were 94.1%, 90.7%, and 87.5%, respectively [38]. In multivariate analysis, male sex, older age at surgery, recurrent meningioma, skull base location, peritumoral edema, WHO II–III, perioperative medical or surgical complications, and a low postoperative KPS score were independent risk factors for increased patient mortality [38]. Regarding recurrence, 62 patients (13.0%) recurred during follow-up, and the 3-year, 5-year, and 10-year recurrence-free survival rates were 93.1%, 89.4% and 79.1%, respectively. Older age, higher age-adjusted Charlson comorbidity index, disease recurrence, involvement of the motor cortex, WHO II–III pathology, and subtotal resection are independent predictors of recurrence [38].

In elderly patients, a recent review and meta-analysis showed that microsurgical resection can achieve a higher proportion of total resection in patients over 80 years old, but the perioperative mortality rate is about 5%, and the surgical risk is relatively high in this population [39]. The study also pointed out that preoperative KPS and American Society of Anesthesiologists (ASA) scores are closely related to postoperative outcomes, and poor function and high anesthesia risk predict higher complications and mortality rates [39]. These findings indicate that perioperative risk control is relatively important for the management of meningiomas in elderly individuals, especially in older patients.

Recently, frailty has been an important prognostic factor in elderly patients with meningioma. Frailty is not age, but a multidimensional state, characterised by low physical resources, reduced stress tolerance, and increased vulnerability. Tariciotti et al.’s [12] frailty-adjusted risk stratification model shows that frailty is closely related to long-term postoperative functional outcomes, quality of life, and complication risk. Therefore, the prognostic assessment of elderly meningioma should shift from the traditional “age + pathology” model to a more comprehensive framework that encompasses age, function, frailty, tumor biology, and treatment methods.

## 5. Management Strategies

### 5.1 Conservative Management

Among elderly patients with meningiomas, conservative treatment is not passive in some cases, but an active strategy [6,21]. Neuroimaging has become more common, and more asymptomatic small meningiomas have been found in the elderly; many studies have shown that these lesions grow slowly or remain stable during follow-up [40,41,42]. Immediate invasive intervention for such patients may not confer significant benefit, but it will also carry unnecessary risks that may affect quality of life.

Several studies have shown that radiological features, such as maximum diameter ≤3 cm, calcification, female sex, low T2-weighted signal, no obvious peritumoral edema, etc., are associated with a lower risk of progression [41,42,43]. Active surveillance is often a reasonable initial option for patients who are very elderly, have high comorbidities, or limited life expectancy. Especially for patients over 80 years old, minor tumor growth may not have clinically meaningful consequences for their remaining lifespan, so initial observation is often a pragmatic approach [17,21,39].

However, conservative management does not mean ignoring it. Some elderly patients’ meningiomas may grow relatively rapidly, leading to increased peritumoral edema or slowly progressive symptoms, especially in those with atypical histology, irregular borders, or proximity to key neurovascular structures [23,44]. If observation is chosen, a structured follow-up needs to be established, including regular MRI examinations, neurological assessments and symptom monitoring. Dynamic assessment of growth rate, anatomical location, potential functional risks and patient preferences usually provides more information than just measuring tumor diameter. Existing evidence also shows that performing preventive surgery on asymptomatic elderly patients may increase the risk of neurological deterioration and other adverse events [45,46], which further supports monitoring as a preventive measure for low-risk lesions.

To facilitate clinical application, we have compiled the imaging features that can be used to guide management stratification (Table 1, Ref. [47,48]). Findings supporting active surveillance include: small maximum tumor diameter (≤3 cm), the presence of calcifications, low T2 signal intensity, absence of peritumoral edema, no evidence of definite growth on serial imaging follow-up, and a location not adjacent to critical neurovascular structures. Indications supporting active intervention (such as surgical resection, stereotactic radiosurgery, or fractionated radiation therapy) include: radiologically confirmed tumor growth, peritumoral edema, T2 hyperintensity, absence of calcification, large maximum tumor diameter (>3 cm), proximity to functional cortical areas or major vascular structures, the presence of significant mass effect, and progressive symptoms. It is important to emphasize that no single imaging feature is decisive; the final management decision should be based on a comprehensive evaluation of imaging findings, clinical symptoms, the patient’s frailty, comorbidities, and the patient’s explicit treatment preferences.

**Table 1.** **Imaging and clinical features guiding management stratification in elderly meningioma patients**.

Feature	Favours active surveillance	Favours active intervention
Maximum tumor diameter	≤3 cm	>3 cm
Growth on serial imaging	No documented growth	Documented growth (tumor diameter increases >3 mm/year or tumor volume increases >15–20%/year) [47]
Calcification	Present	Absent
T2 signal	Hypointense	Hyperintense
Peritumoral edema	Absent	Present
Mass effect	Absent	Present
Location	Non-eloquent, remote from critical structures	Adjacent to eloquent cortex, cranial nerves, or major vessels
Clinical symptoms	Asymptomatic, incidentally detected	Progressive neurological/cognitive deficits, seizures, raised ICP
WHO grade (pathology/suspicion)	Grade I, classic imaging features	Grade II–III features, irregular margins, brain invasion suspected
Suggested management	Active surveillance (6-month, then annual MRI) [47]	Surgery/SRS/Fractionated RT/MDT discussion
Avoid aggressive intervention if	—	Age ≥ 85 years old and highly frail, unable to withstand intervention treatment [48]

WHO, World Health Organization; MRI, magnetic resonance imaging; ICP, intracranial pressure; SRS, stereotactic radiosurgery; RT, radiation therapy; MDT, multidisciplinary team.

### 5.2 Surgical Treatment

Surgical resection is an important treatment for elderly patients with meningiomas, especially for those with obvious symptoms, large masses, progressive neurological deficits, or definite tumor growth during follow-up [6]. Traditionally, the goal of meningioma surgery is to maximize tumor resection while preserving neurological function, relieving the mass, improving symptoms, and reducing the risk of recurrence [6]. However, whether gross total resection is the primary goal remains controversial in elderly patients.

Multiple studies have shown that the postoperative mortality rate and complication rate of elderly patients after meningioma surgery are higher than those of young patients [23,24,36,49]. Meta-analysis evidence shows that older people are more prone to neurological and systemic complications, a higher ASA grade is a key precursor of neurological complications, and advanced age is more closely associated with systemic adverse events [23]. Bateman et al. [19] reported that the postoperative mortality rate of patients over 70 years old is about 4%, nearly 4 times that of young patients, and the risk of poor prognosis in patients over 80 years old increases again. Diaz et al. [39] also pointed out that even if 88% of patients ≥80 years old underwent total resection, the perioperative mortality rate remained about 5%. Overall, these research findings indicate that older adults, especially those who are very elderly, are more susceptible to the effects of surgical stress.

At the same time, when patients are carefully selected, elderly individuals may still derive substantial benefit from surgery. Several studies have shown that in patients with relatively high preoperative KPS, no severe neurological deficits, controllable comorbidities, and surgically accessible tumor locations, surgery can provide effective symptom relief and acceptable long-term outcomes [37,50]. These findings indicate that age alone should not be considered an absolute contraindication to surgery. Rather, the key determinant of surgical value is whether the patient is likely to achieve net clinical benefit.

The surgical approach to elderly meningiomas has changed from “maximum resection” to “maximum safety benefit”. For some tumors located at the skull base, within the paranasal sinuses, or close to neurovascular structures, complete resection may increase the risk of complications and functional decline. Elderly patients usually recover poorly. For elderly patients, partial resection is more reasonable. Partial resection can reduce operative duration, blood loss, and anesthesia burden. The residual tumor may not cause clinical problems during the patient’s remaining life, and, if necessary, stereotactic radiosurgery (SRS) can be used for control [6,51]. This concept conforms to the “residual coexistence” concept, and a function-oriented elderly staging method has also been developed.

In addition, Preoperative risk stratification tools have certain value for surgical decision-making in elderly patients with meningiomas. KPS, ASA, SKALE (Sex, Karnofsky, ASA score, Location, Edema) scores and frailty assessments can help identify patients who may benefit from aggressive surgery [52,53,54,55]. Especially in patients aged ≥80 years, functional status and systemic risk factors other than age may predict postoperative outcomes better than age alone. Therefore, the extent of resection in elderly patients should not be determined by age alone, but by the anticipated balance between tumor control and treatment-related morbidity. In fit older adults with good baseline function and surgically accessible convexity or non-eloquent lesions, gross total resection may still be an appropriate goal when it can be achieved with acceptable risk. In contrast, for skull base, petroclival, cavernous sinus, or other neurovascularly complex meningiomas—particularly in frail, very elderly, or functionally vulnerable patients—planned subtotal resection for decompression and symptom relief, followed by surveillance or stereotactic radiosurgery when appropriate, may provide a more favorable risk–benefit profile.

### 5.3 Radiotherapy

In the comprehensive treatment of elderly patients with meningiomas, radiotherapy has become increasingly important, especially in cases where craniotomy is not suitable, postoperative residual, recurrent or high-grade meningiomas [6,51]. The common methods are stereotactic radiosurgery and fractionated radiotherapy. Generally speaking, stereotactic radiosurgery is targeted at lesions with clear borders, small volume, and far from key structures; fractionated radiotherapy is targeted at larger tumors, obvious peritumoral edema, and tumors close to nerves, the brainstem, or functional areas [56,57].

Current evidence indicates that radiation therapy (RT) achieves a relatively high local control rate, with tumor control rates ranging from 90% to 95% [51,58,59]. For skull base meningiomas, cavernous sinus lesions or elderly patients with high surgical risks, radiotherapy is particularly attractive [57]. Some studies suggest that primary SRS may improve the progression-free survival (PFS) of incidental meningiomas, and the risk of secondary neurological deficits is relatively low [60]. Therefore, among elderly patients, when their tumors show interval growth on imaging but are not suitable for open surgery, SRS may provide a less invasive alternative.

In the setting of adjuvant therapy, radiotherapy has a more obvious effect on incompletely resected and high-grade meningiomas. WHO I meningiomas usually do not have routine adjuvant radiotherapy after gross total resection. Still, in cases of recurrence/residual tumors in complex areas, adjuvant/salvage radiotherapy may be required [61]. For WHO II meningiomas, adjuvant radiotherapy is often recommended after subtotal resection, and the role of adjuvant radiotherapy after gross total resection is controversial; evidence suggests that it may improve local control and progression-free survival, but overall survival benefits are not always evident [62,63,64]. WHO III meningiomas usually use adjuvant radiotherapy, but for elderly patients, the degree of benefit needs to be individually adjusted according to the extent of resection, functional status, and treatment goals [65,66,67].

It should also be acknowledged that radiotherapy in elderly patients raises specific concerns, including cognitive decline, radiation necrosis, and exacerbation of peritumoral edema [68,69,70]. Although some evidence suggests that the radiation doses commonly used for meningioma may have a limited cognitive impact, careful evaluation remains necessary in patients with preexisting cognitive impairment, extensive white matter disease, or marked frailty. Overall, Stereotactic radiosurgery should not be regarded as a universal alternative to surgery for elderly patients. It is most suitable for selected small tumors, residual or recurrent lesions, patients at high surgical risk, or meningiomas located in anatomically complex regions. In contrast, large tumors with marked mass effect, significant peritumoral edema, progressive neurological impairment, or those requiring decompression or histological diagnosis are generally not suitable for SRS. They are better managed through surgery or combined approaches.

Therefore, clinical decision-making for elderly patients with meningiomas should be driven by benefit rather than by age thresholds. In practice, treatment options should be determined by addressing several key questions: (1) Is the tumor symptomatic or showing radiological progression? (2) Is there an associated mass effect or progressive edema? (3) Is a definitive diagnosis or decompression required? (4) Is the lesion resectable, and are the neurological and systemic risks acceptable? (5) Does the patient have sufficient functional reserve, cognitive status, and overall health to derive meaningful benefit from the intervention? For asymptomatic, low-risk lesions without obvious growth or mass effect, observation is generally the preferred approach. Surgery is indicated when symptoms, growth, edema, mass effect, or the need for decompression/histological diagnosis are present, and the surgical risk is acceptable. SRS is indicated for small-volume tumors, residual or recurrent lesions, anatomically complex locations, or patients with increased surgical risk. When tumor size or proximity to the optic nerve, cranial nerves, or other critical structures makes a single SRS session unsuitable, fractionated SRS is preferred. When curative surgery is unlikely to improve functional outcomes, combined strategies—such as SRS following limited resection or fractionated SRS—may be particularly useful (Table 2).

**Table 2.** **Treatment selection in elderly patients with meningioma**.

Clinical scenario	Observation	Surgery	SRS	Fractionated RT	Combined strategy
Asymptomatic, small, low-risk lesion, no growth	Preferred	Usually not first-line	Consider only selected cases	Rarely needed	No
Symptomatic tumor with mass effect	Usually not sufficient	Preferred	Usually unsuitable	Usually unsuitable as first-line	Possible after limited surgery
Documented growth on serial imaging	Consider if frail and slow-growing	Consider if accessible and patient fit	Suitable in selected small/moderate lesions	Suitable near critical structures or larger lesions	Consider
Skull base/cavernous sinus/petroclival lesion	Selected cases	Often limited/debulking rather than aggressive GTR	Often useful for small residual or primary selected lesions	Often useful	Frequently relevant
Large lesion with marked edema	Not preferred	Often required for decompression	Generally less suitable	Selected only, depending on goal	Often reasonable
Severe frailty, advanced cognitive impairment, high dependency, limited life expectancy	Often preferred	Usually limited benefit	Case-by-case	Case-by-case	Usually not appropriate unless symptom palliation expected

GTR, gross total resection.

## 6. Discussion

### 6.1 Increasing Disease Burden but Insufficient High-Quality Evidence

Meningioma in older adults has become one of the most common challenges in neuro-oncology and geriatric neurosurgery, yet a substantial gap remains between clinical demand and the available evidence base [6,71,72]. On the one hand, the number of diagnosed cases—particularly incidental lesions—continues to rise as a result of population aging and the widespread use of neuroimaging [41,71,72,73,74]. On the other hand, high-quality prospective studies and standardized decision-making criteria remain limited, and most currently available evidence derives from retrospective single-centre studies with considerable heterogeneity in patient selection and outcome definitions [6,17,23,71,75,76]. As a result, substantial variability persists in the management of similar patients across institutions.

Importantly, treatment goals in elderly patients differ from those in younger adults [6,12,38,71]. Whereas management in younger patients often emphasises long-term tumor control and minimisation of recurrence, elderly patients are more likely to prioritise perioperative safety, preservation of neurological function, cognitive stability, and maintenance of independent living [12,38,71]. Therefore, treatment paradigms derived from the general adult meningioma population cannot be directly extrapolated to older patients without modification. Future progress in this field will depend on the development of an evidence framework more closely aligned with geriatric principles and function-oriented outcomes [6,12,71].

### 6.2 From an Age-Centered to a Benefit-Centered Model

Chronological age has long been regarded as a major determinant of treatment intensity, particularly in patients aged 80 years and above [19,75,77,78]. However, growing evidence suggests that age itself is neither the sole nor the most decisive predictor of adverse outcomes [12,13,17,38,71,76]. Rather, outcomes are more strongly influenced by clinical factors commonly associated with advanced age, including comorbidity burden, frailty, preoperative KPS, cognitive status, nutritional condition, and functional dependence [12,13,17,38,76]. Frailty and nutritional status reflect a patient’s overall condition and physical fitness, which can affect surgical tolerance and overall recovery. The threshold for aggressive surgery should be significantly higher for such patients, as treatment-related morbidity may outweigh the potential benefits of tumor control; cognitive status determines a patient’s treatment prognosis; such patients face a higher risk of poor functional recovery and loss of independence, so aggressive treatment should be approached with greater caution; postoperative delirium has significant practical implications for elderly patients, as it may lead to long-term cognitive and functional decline, severely affecting treatment outcomes; this should be a key consideration during preoperative risk-benefit discussions; social support and functional dependence influence a patient’s postoperative care; a lack of support after surgery may prevent a return to independent living. Therefore, treatment decisions regarding social support should not be based solely on the tumor itself.

This distinction has important practical implications [12,38]. Some very elderly patients may still derive substantial benefit from active treatment if they have good baseline function, clear tumor-related symptoms, relatively superficial lesions, and acceptable surgical risk [17,36,38,75,79]. Conversely, even patients who are not extremely old may derive limited benefit if they already have pronounced frailty, functional dependence, or severe systemic disease [12,38,76]. Decision-making in elderly meningioma should therefore move away from an age-centered model and toward a benefit-centered one, focusing on whether a given intervention is likely to provide meaningful functional and survival benefits rather than relying on age thresholds alone [12,38].

### 6.3 Incidental Meningioma in Older Adults Has Changed the Logic of Management

The elderly should not be viewed as a homogeneous population. Definitions of “the elderly” vary in the literature; some studies use 65 years as the cutoff, while others use 70 or 80 years [17,71,75]. However, in clinical practice, patients aged 65, 75, and 85 differ significantly in terms of physiological reserve, life expectancy, burden of comorbidity, and perioperative tolerance; therefore, grouping them together may obscure clinically significant differences [12,38,71]. For the purposes of this study, we further divided older adults into two categories: the elderly (ages 65–79) and very elderly (≥80). This stratification is clinically meaningful because treatment goals differ significantly between these groups. For patients aged 65–79, long-term tumor control and minimizing recurrence remain relevant goals alongside functional preservation. For patients aged ≥80 years, the primary goals are almost exclusively symptom relief, maintenance of quality of life, and avoidance of harm; aggressive intervention is rarely justified unless the patient has exceptional baseline function, significant symptoms, and is a candidate for surgical resection [39,71,75,78]. This heterogeneity is particularly important in the management of incidentally discovered meningiomas.

In the era of modern neuroimaging, an increasing proportion of meningiomas in older adults are detected incidentally, fundamentally altering the logic of clinical decision-making [41,73,74]. Historically, meningiomas were more often diagnosed only after producing overt symptoms, making the indication for treatment relatively straightforward. By contrast, many contemporary patients are identified while asymptomatic or only mildly symptomatic, requiring a different management framework in which the central question is no longer how to treat, but whether treatment is needed at all [41,73,74].

Available evidence generally supports an observation-first strategy for small lesions located outside critical functional areas that are densely calcified, T2-hypointense, and without peritumoral edema [41,71,74]. This approach reflects not only the typically indolent growth pattern of such tumors but also that, in older adults, limited life expectancy, comorbidity burden, and treatment-related risks mean that radiological detection alone does not necessarily justify immediate intervention. Nevertheless, incidental discovery should not be equated with uniformly low risk [41,74]. Continued tumor growth, progressive edema near eloquent structures, or the development of new focal symptoms should prompt reassessment of the timing of intervention [6,41,71,74]. Thus, in older adults with incidental meningioma, the key clinical issue is not simply whether to observe or treat, but when observation should transition to active management [41,74].

Therefore, in practical clinical practice, for small to medium-sized, incidentally discovered, asymptomatic, and low-risk meningiomas, it is recommended to adopt an initial strategy of active monitoring through regular MRI. The monitoring interval should be personalized: MRI examination should be conducted every 6 months in the first year, and if stable, once a year, and reevaluated based on growth, new symptoms, or imaging changes. For meningiomas that are symptomatic or growing, accompanied by space-occupying effects, edema, or progressive neurological/cognitive symptoms, this situation requires a positive risk-benefit balance. At present, microsurgical resection may achieve ultimate control for elderly patients with fully functional and surgically resectable lesions. For patients with high surgical risk but substantial mass effects, staged or palliative methods such as subtotal resection combined with SRS can be considered. For residual, recurrent, or WHO II–III meningiomas, clinical decision-making should be guided by the speed of previous tumor recurrence, the degree of previous treatment, the presentation status, as well as the values and nursing goals of the patient and family.

### 6.4 Reframing Surgical Goals and Integrating Surgery With SRS

Conventional meningioma surgery has traditionally emphasized Simpson grade I or II resection, when feasible, to reduce recurrence [6,17]. In elderly patients, especially the very elderly, this goal should be reconsidered in light of overall patient benefit rather than extent of resection alone [12,17,71,75]. Whereas recurrence control is often the dominant endpoint in younger patients, outcomes such as perioperative mortality, neurological deterioration, cognitive decline, long-term dependence, and loss of independent living may be of greater immediate clinical relevance in older adults [12,38,75,78]. Accordingly, the surgical objective in elderly meningioma may be better defined as maximizing safe benefit rather than maximizing safe resection [12,17,71,75]. Treatment success should therefore be judged not only by the extent of resection, but also by symptom relief, preservation of function, recovery potential, and quality of life [12,38].

Within this framework, surgery and SRS should be viewed not as competing options, but as complementary modalities along a therapeutic continuum [6,42,71]. Surgery provides rapid decompression, tissue diagnosis, and symptomatic relief, whereas SRS offers a less invasive means of controlling residual, recurrent, or surgically high-risk lesions [42,71]. This combined approach is particularly relevant for skull base lesions, tumors invading the paranasal sinuses, and lesions adjacent to major neurovascular structures, where aggressive gross total resection may increase the risk of permanent neurological injury and where oncological gains may not justify the functional cost [6,17,42,71]. In such settings, limited decompression or subtotal resection followed by SRS may provide a more favorable balance between tumor control and treatment burden, especially in very elderly or frail patients, and is consistent with a function-first, low-burden management strategy in geriatric neurosurgery [12,17,39,42].

### 6.5 Cognitive and Quality-of-Life Outcomes Should Receive Greater Attention

Most current studies on meningioma in elderly patients continue to focus primarily on traditional endpoints such as mortality, complication rates, length of hospital stay, and recurrence, while giving insufficient attention to the outcomes that matter most to older patients [12,75,76,78]. For many very elderly individuals, the ability to walk, eat, use the toilet, communicate, and continue living at home independently after treatment may be more important than achieving radiological gross total resection [12,38]. Similarly, postoperative cognitive trajectories, long-term delirium, and the need for institutional care may better reflect the real value of treatment than local tumor control alone [12,13].

Accordingly, future studies should place greater emphasis on functional independence, cognitive outcomes, symptom relief, rehabilitation requirements, and patient-reported outcomes [12,38]. Only when the study design is aligned with the priorities of elderly patients can research findings more effectively inform clinical practice.

### 6.6 Limitations of the Current Evidence Base

Although the literature on elderly meningioma has expanded substantially in recent years, several important limitations remain [17,23,71,75,76]. First, most available studies are retrospective and therefore susceptible to selection bias, since patients selected for surgery are often healthier at baseline, potentially leading to an overestimation of surgical benefit [17,23,76]. Second, definitions of “elderly” vary across studies, and there is considerable heterogeneity in tumor location, pathological grade, and perioperative management, which complicates direct comparisons across cohorts [23,71,75,76]. Third, many studies have limited follow-up duration and may therefore underestimate long-term cognitive decline, functional deterioration, and late recurrence [12,38,76]. Finally, inconsistencies in the definitions of complications and functional outcomes reduce cross-study comparability and limit the robustness of meta-analytic conclusions [75,76].

Accordingly, two interpretive extremes should be avoided: excessive conservatism based solely on the elevated risk associated with advanced age, and indiscriminate expansion of surgical indications based on reports suggesting that surgery is “acceptable” in selected elderly patients [12,17,71,75,78]. In the setting of an incomplete evidence base, individualized, multidisciplinary, and patient-centered decision-making remains essential [6,12,71].

## 7. Future Perspectives

In the future, we believe that research and clinical practice focused on meningiomas in older people should receive greater attention. For example, a risk stratification model specifically designed for elderly patients should be developed as soon as possible to assess patient prognosis and help clinicians make more accurate decisions. Although traditional indicators such as age, KPS, and ASA scores are useful, they are not sufficient to fully capture treatment risks. Future models should incorporate the above factors and combine frailty, nutritional status, cognitive function, radiological features, and potential molecular pathological markers. Secondly, strengthen interdisciplinary collaborative management, diagnosis and treatment. The diagnosis and treatment of elderly meningioma patients involve not only neurosurgeons but also anesthesiologists, geriatricians, rehabilitation specialists, neuroradiologists, radiation oncologists, and professional nursing teams. Multidisciplinary discussions may contribute to more comprehensive risk assessment and optimization of treatment decisions. Thirdly, future research should place greater emphasis on endpoints such as functional prioritisation, cognitive outcomes, and quality of life, rather than just focusing on issues such as survival and recurrence. For elderly patients, maintaining acceptable daily functioning after diagnosis and treatment is usually more meaningful than prolonging survival alone. In addition, minimally invasive diagnosis and combined treatment strategies are worth further research, including limited decompression, subtotal resection combined with SRS, and more refined perioperative management. Finally, with the accumulation of research evidence, standardized diagnosis and treatment recommendations for specific age groups should be developed based on existing guidelines for meningiomas to improve the consistency of treatment concepts and clinical practices across centres.

## 8. Conclusions

Global population aging has led to an upward trend in the incidence, detection and management of meningiomas in older people. Elderly patients with meningiomas differ from younger patients in terms of tumor biology, clinical manifestations, detection, and treatment tolerance, and require more individualized care. For asymptomatic, smaller and radiologically low-risk lesions, active surveillance is usually a reasonable and safe strategy. For tumors with obvious symptoms, mass effect, or persistent radiological progression, surgery is an important treatment option; however, the treatment goal should focus more on relieving symptoms, preserving neurological function, and improving quality of life, rather than pursuing complete resection. Radiotherapy, especially stereotactic radiosurgery, has important comprehensive value in the management of patients with high surgical risk, anatomically complex lesions and postoperative residual disease. Overall, decision-making for elderly meningioma patients needs to shift from age-centric to benefit-centric, integrating functional status, frailty, comorbidities, tumor characteristics, and individual treatment goals. More high-quality prospective studies focused on elderly and very elderly patients should be conducted to clarify the benefits of treatment strategies and advance precise, standardized, patient-centered meningioma management in this growing population.
